# Supplementary material for: Development characteristics of the rock fracture field in strata overlying a mined coal seam group
Source: PLoS One. 2022 Oct 5;17(10):e0268955. doi: 10.1371/journal.pone.0268955 (PMC9534389; doi:10.1371/journal.pone.0268955)
Supplement: S1 File — (DOCX) [file pone.0268955.s001.docx]

**The data set of all parameters needed in the paper：**

1. Taking the fully mechanized top coal caving face F15.16-24130 in the Pingmei No. 10 coal mine as the research object, The F_15.16_ coal seam was the mined coal seam. The direct roof is sandy mudstone plus a thin layer of carbonaceous mudstone and coal seam F14, which is relatively hard, with a thickness of 11–18 m. The main roof is grayish white fine to medium grained sandstone with a thickness of 10.9 m. The thickness of coal seam F15 is about 1.9 m, the thickness of coal seam F16 is about 1.5 m, the thickness of composite layer F15.16 is 2.8–4.2 m, generally about 3.5 m, and the dip angle of the coal seam is 6–13°, with an average of 9.5°. The firmness coefficient of coal seam F15 is f = 2–3. The thickness of the gangue between F15 and F16 is 0.2–0.7 m, and it gradually becomes thinner from outside to inside. The direct bottom is sandy mudstone with a thickness of 7.2–8.5 m. It is below coal seam F17. The old bottom is sandy mudstone with a thin layer of fine sandstone and limestone, and the thickness is >10 m.

2. Physical parameters of coal and rock mass

| Material Science | thickness (m) | density (kg·m^-3^) | tensile strength (MPa) | cohesion (MPa) | bulk modulus (GPa) | shear modulus (Pa) | internal friction angle (°) |
| --- | --- | --- | --- | --- | --- | --- | --- |
| Fine to medium grained sandstone | >18 | 2600 | 6.5 | 13.4 | 57 | 3.40E+10 | 36 |
| Fine to medium grained sandstone | 1.8 | 2600 | 6.5 | 13.4 | 57 | 3.40E+10 | 36 |
| Coal seam F_14_ | 0.4 | 2600 | 6.5 | 13.4 | 57 | 3.40E+10 | 36 |
| Sandy mudstone | 13 | 2400 | 4.1 | 6.8 | 46 | 2.42E+10 | 46 |
| Coal seam F_15.16_ | 3.5 | 1410 | 1.8 | 2.1 | 25 | 1.48E+10 | 25 |
| Sandy carbonaceous mudstone | 2.8 | 2400 | 4.1 | 6.8 | 46 | 2.42E+10 | 46 |
| Coal seam F_17_ | 2.5 | 1410 | 1.8 | 2.1 | 25 | 1.48E+10 | 25 |
| Gray packsand | 6 | 2600 | 7.8 | 15.4 | 62 | 3.74E+10 | 45 |
| Sandy mudstone | 4 | 2400 | 4.1 | 6.8 | 46 | 2.42E+10 | 46 |
| Limestone L1 | 4 | 2400 | 2.8 | 3.7 | 38 | 2.33E+10 | 28 |
| Coal line | 0.3 | 2400 | 2.8 | 3.7 | 38 | 2.33E+10 | 28 |
| Sandy mudstone | >10 | 2400 | 4.1 | 6.8 | 46 | 2.42E+10 | 46 |

3. Geostress parameters：

| Maximum principal stress | | | Intermediate principal stress | | | Minimum principal stress | | |
| --- | --- | --- | --- | --- | --- | --- | --- | --- |
| size (MPa) | direction (°) | angle (°) | size (MPa) | direction (°) | angle (°) | size (MPa) | direction (°) | angle (°) |
| 34.32 | −157.6 | −16.9 | 22.19 | −141 | 71.4 | 18.3 | −66.6 | −4.8 |

4. Layout of monitoring line and parameters of measuring points

| Serial number of observation lines | | Rock properties at the observation lines | | Horizon property | Measuring points | | Distance from coal seam floor (m) |
| --- | --- | --- | --- | --- | --- | --- | --- |
| 1 | Fine to medium grained sandstone | | Key layer | | 100 | 22.5 | |
| 2 | Fine to medium grained sandstone | | Coal seam roof | | 100 | | 13.2 |
| 3 | Sandy carbonaceous mudstone | | Interlayer between coal seams F_15.16_ and F_17_ | | 100 | | 3.9 |
| 4 | Gray packsand | | Coal seam floor | | 100 | | 0 |

5. In addition, I also give the data used to construct the curve in Figure 6, Figure 7, Figure 12 and figure 13 in the *excel* file(Named *All data of stress + displacement* ). These data are obtained through numerical simulation analysis and do not belong to the direct data constituting the paper. I think the minimum data set should be the direct data constituting the paper. According to these minimum data sets, the indirect data in Figure 6, Figure 7, Figure 12 and figure 13 can be deduced. Therefore, In my opinion, the data required in Figure 6, Figure 7, Figure 12 and figure 13 do not belong to the minimum data set.
