# Supplementary material for: Development characteristics of the rock fracture field in strata overlying a mined coal seam group
Source: PLoS One. 2022 Oct 5;17(10):e0268955. doi: 10.1371/journal.pone.0268955 (PMC9534389; doi:10.1371/journal.pone.0268955)
Supplement: Attachment — Submitted filename: Response to Reviewers.docx [file pone.0268955.s003.docx]

Dear editor and reviewers:

Thank you for your letter and the reviewers’ comments on our manuscript entitled " Development Characteristics of the Rock Fracture Field in Strata Overlying a Mined Coal Seam Group". Those comments are very helpful for revising and improving our paper, as well as the important guiding significance to other research. We have studied the comments carefully and made corrections which we hope meet with approval. The main corrections are in the manuscript and the responds to the reviewers’ comments are as follows (the replies are highlighted in blue ).

I checked the list of all references, and I can guarantee the integrity and correctness of each reference.

**Replies to the reviewers’ comments:**

**Response to reviewers**

**Reviewer #1:** The article is innovative and can be published.The English level of the article meets the requirements.The number of references is relatively small, and it can be appropriately increased to about 25 references.

**Response:** **References have been added as required. At present, there are 26 references, and the newly added references have been marked in blue font.**

**Response to Reviewer #2:**

1. The conclusion should be concise and rewritten.

**Response: The conclusion part has been condensed again with concise language**

2. The letters or parameters in Figure 1 should be clear.

**Response:Figure 1 has been modified as CAD source drawing, and all its letters and parameters are clearly visible**

3. For numerical simulation, why did not the effect of fault structure on rock movement be considered?

**Response:Because the mechanical strength of the rock stratum at the fault zone is lower than that of the surrounding rocks, and the fractures are relatively easy to develop, the evolution law of rock fractures at the fault zone can not truly reflect the evolution law of overburden fractures affected by mining under the mining conditions of coal seams. In addition, there is no fault structure in the working face within the research scope of the project group, However, the influence of fault structure on the evolution of overburden fractures will be considered in the subsequent research process.**

4. The basis for the values of various moduli in Table 1 should be explained in detail.

**Response:** **References 20 and 21 have been added to the paper. These two references are the project reports of the research group. The acquisition process of various data in Table 1 is described in these two references.**
